# From Omics to Multi-Omics: A Review of Advantages and Tradeoffs

**DOI:** 10.3390/genes15121551

**Published:** 2024-11-29

**Authors:** C. Nelson Hayes, Hikaru Nakahara, Atsushi Ono, Masataka Tsuge, Shiro Oka

**Affiliations:** 1 Department of Gastroenterology, Graduate School of Biomedical & Health Sciences, Hiroshima University, Hiroshima 734-8551, Japan; atsushi-o@hiroshima-u.ac.jp (A.O.); tsuge@hiroshima-u.ac.jp (M.T.); oka4683@hiroshima-u.ac.jp (S.O.); 2 Department of Clinical and Molecular Genetics, Hiroshima University, Hiroshima 734-8551, Japan; hnkhr@hiroshima-u.ac.jp; 3 Liver Center, Hiroshima University, Hiroshima 734-8551, Japan

**Keywords:** bioinformatics, epigenomics, transcriptomics, proteomics, metabolomics, lipidomics, glycoinformatics, single-cell RNA sequencing, spatially resolved transcriptomics

## Abstract

Bioinformatics is a rapidly evolving field charged with cataloging, disseminating, and analyzing biological data. Bioinformatics started with genomics, but while genomics focuses more narrowly on the genes comprising a genome, bioinformatics now encompasses a much broader range of omics technologies. Overcoming barriers of scale and effort that plagued earlier sequencing methods, bioinformatics adopted an ambitious strategy involving high-throughput and highly automated assays. However, as the list of omics technologies continues to grow, the field of bioinformatics has changed in two fundamental ways. Despite enormous success in expanding our understanding of the biological world, the failure of bulk methods to account for biologically important variability among cells of the same or different type has led to a major shift toward single-cell and spatially resolved omics methods, which attempt to disentangle the conflicting signals contained in heterogeneous samples by examining individual cells or cell clusters. The second major shift has been the attempt to integrate two or more different classes of omics data in a single multimodal analysis to identify patterns that bridge biological layers. For example, unraveling the cause of disease may reveal a metabolite deficiency caused by the failure of an enzyme to be phosphorylated because a gene is not expressed due to aberrant methylation as a result of a rare germline variant. **Conclusions**: There is a fine line between superficial understanding and analysis paralysis, but like a detective novel, multi-omics increasingly provides the clues we need, if only we are able to see them.

## 1. Introduction

With few exceptions, each cell in the human body contains its own complete copy of the genome, but individual cells vary enormously in terms of morphology, composition, and longevity. The Human Cell Atlas defines at least 300 distinct cell types [1], and cell morphology and metabolic activity can vary over time and spatial orientation even among cells of the same type. Characterizing these differences is essential within the field of medicine, especially in terms of drug development. Despite sharing the same underlying DNA, the repertoire of genes available to cells of each type is limited as a result of epigenetic changes such as methylation and acetylation. Many genes are only expressed in the presence of external growth factors, and the expression of numerous genes is tightly regulated in response to environmental conditions within and outside of the cell. Measuring gene expression (genomics, transcriptomics, and epigenomics) within homogenous samples provides valuable insight into the cell, but it does not provide a complete picture. Measuring the expression level of a protein-coding gene is not the same as quantifying the translated protein (proteomics), and the function of many proteins is further influenced by post-translational modifications such as phosphorylation (phosphoproteomics) and glycosylation (glycomics and glycoinformatics). Furthermore, the presence of an enzyme is not evidence of its activity, and the presence of expected metabolites or lipids should be confirmed directly (metabolomics and lipidomics). Given this vast range of available omics methods, which one(s) should be selected? The flippant answer, as many as possible, is often impractical due to the combinatorial costs as well as technical limitations, such as sample volume or quality.

**Figure 1:**
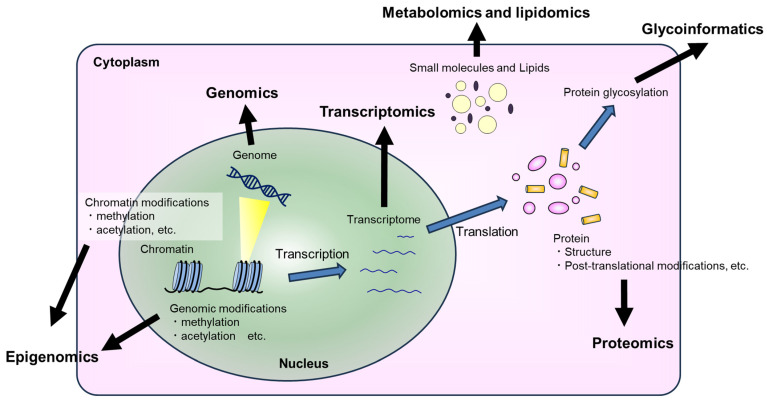
There are numerous omics methods, but each provides insight into a distinct aspect of the biological state of a sample. Genomics and epigenomics focus on the sequence and accessibility of the genetic material, whereas transcriptomics, proteomics, metabolomics, and glycoinformatics examine the expression levels, post-translational modifications, and biochemical activity of proteins and other biomolecules under different conditions.

In this review, we set out some of the advantages, challenges, and trade-offs across the full range of multi-omics research (Figure 1). Although the goal of multi-omics research is to synthesize a more comprehensive understanding by incorporating data representing orthogonal perspectives of cellular processes, this should not imply that each distinct type of omics data contributes equally or is equally mature. The review is structured roughly in order of the most mature (genomics) to the least mature (metabolomics/lipidomics), followed by a discussion of the challenges in integrating diverse omics data sets.

## 2. Methods and Tools for Single-Omics Analysis

### 2.1. Genomics

As the National Human Genome Research Institute proclaims, the Human Genome Project (HGP) was “one of the greatest scientific feats in history”. Launched in 1990 and completed in 2003, the HPG remains one of the largest international scientific collaborations ever undertaken, spanning six nations at a cost of USD 2.7 billion. The pioneering sequencing efforts fueled innovation at every level, from sequencing technologies to data storage and software development, and the human reference sequence continues to form the foundation of modern bioinformatics. However, the project ended in 2003 with the lingering sense that the human genome laid bare was not the open book some had expected [2]. Annotation of the human genome is an ongoing, long-term effort and has been updated numerous times. The latest genome assembly is T2T-CHM13v2.0 [3], although Genome Reference Consortium Human Build 38 (GRCh38 or hg38), first released in 2013, and even Build 37 (GRCh37 or hg19), first released in 2009, are still widely used, partly due to incompatibility between the coordinates used among the major releases.

Sequencing technologies are often grouped into first-generation, second- or next-generation, and third-generation sequencing technologies that vary tremendously in terms of read length, cost, accuracy, and difficulty of analysis (summarized in Table 1) [4,5].

#### 2.1.1. Hierarchical Shotgun Sequencing

The human genome was sequenced using hierarchical shotgun sequencing, a laborious process in which the genome was broken into 150–200 kb fragments and cloned into bacterial artificial chromosomes. Amplified sequences were then further fragmented and sequenced across multiple laboratories using Sanger sequencing.

#### 2.1.2. First Generation: Sanger Sequencing

In this first-generation chain termination method, the complementary DNA strand is synthesized with the addition of a small number of fluorescently labeled nucleotides modified to prevent further chain extension. In principle, this should produce chains that terminate at each position along the sequence. The original DNA sequence can therefore be reconstructed by sorting the fragments based on size on an electrophoretic gel and reading the order of the fluorescent labels.

#### 2.1.3. Second/Next Generation: Massively Parallel Sequencing

While the HGP was able to sequence multiple fragments in parallel through careful planning and coordination, this approach has been superseded by massively parallel sequencing (next-generation sequencing), which is capable of generating multiple reads in parallel during the same sequencing run using a method called sequencing by synthesis. Libraries are created by extracting and PCR-amplifying sample DNA, and the addition of each type of nucleotide is monitored during the synthesis of the complementary strand. This method produces a large number of relatively short reads. In paired-end sequencing, overlapping forward and reverse reads with a fixed offset are used to aid assembly.

Although NGS methods improved the speed and cost of sequencing, there is a tradeoff. The large number of short reads requires stringent quality control steps. Reads are exported as FASTQ files that contain not only the sequence but the location of the read on the flow cell as well as a Phred quality score that indicates the probability of an incorrect base call at each position. Tools like FastQC and Trimmomatic [6] are used to remove adapters, exclude chimeric reads, and trim low-quality sequences from the ends. Newly sequenced genomes require reads to be assembled without reference to an existing genome sequencing using methods such as greedy algorithms and De Bruijn graphs. Popular de novo assemblers include ABySS [7], Trinity [8], SPAdes [9], Velvet [10], and SOAPdenovo2 [11].

When a reference genome is already available, tools such as BWA [12] and Bowtie2 [13] are used to align each read to the reference sequence. This problem is computationally challenging due to the large number of reads, the use of reads that are expected to map in the correct orientation on both the forward and reverse strands, the potential for insertions and deletions, and the potential of reads from non-unique regions of the genome to map equally well to two or more locations. Aligned reads are stored in a Sequence Alignment Map (SAM) file, which is normally compressed into an indexed binary format (BAM and BAI files) using SAMtools [14]. BAM files can be visualized using genome viewers such as Integrative Genomics Viewer [15] and Tablet [16], which provide information on the sequencing depth and consensus sequence as well as the CIGAR string for each read. Once reads are aligned to the reference sequence, variant calling is performed using tools such as Bcftools mpileup or GATK HaplotypeCaller, and differences from reference alleles are saved in Variant Call Format (VCF) files. Variant calling is highly dependent on the local alignment at each position, but the Genome Analysis Toolkit provides best practices and tools to improve variant calling accuracy [17].

#### 2.1.4. Third Generation: Long-Read Sequencing

The reference genome published in 2003 was 92% complete, but numerous gaps remained, consisting of long stretches of highly repetitive DNA that could not be sequenced with the technology of the time. The last remaining gaps were only completed in 2022 [3] with the aid of long-read sequencing methods pioneered by Pacific Biosciences and Oxford Nanopore Technology.

##### PacBio Sequencing

The process of assembly works by comparing regions of overlap among reads, but many regions of the genome contain stretches of highly repetitive, palindromic, or structurally complex DNA to which short reads cannot map uniquely, resulting in gaps. Based on technology originally developed at Cornell University, Pacific Biosciences (PacBio) HiFi sequencing offered an elegant solution to this long-standing problem [18]. In single-molecule real-time (SMRT) sequencing, each DNA fragment is circularized by ligating with hairpin adapters and attached to a DNA polymerase fixed to the bottom of a microscopic well called a zero-mode waveguide (ZMW). Early PacBio RS chips contained around 150,000 wells, but this number jumped to over 8 million in the follow-up Sequel IIe system, while the latest Revio SMRT Cells contain over 25 million wells. As four SMRT Cells can be run at the same time, up to 100 million cells can be sequenced simultaneously. Each well is illuminated from below in such a way that only one nucleotide at a time is illuminated as it passes through the polymerase. Each type of nucleotide contains a different fluorescent dye, which is cleaved when the base is ligated to the growing DNA strand, and the detected signal is recorded as a base call. As the circular DNA returns to the starting point, the sequenced subread is expected to contain randomly distributed errors at a rate of about 10–15% per base; however, the template is read at least 8–10 times, and a highly accurate circular consensus sequence read is generated, with an error rate of <0.1%. This system combines high accuracy with long read length, but drawbacks include the high cost of reagents and the longer time and greater computational power required to process the consensus data. Nonetheless, the latest system resolves some of these problems and improves processing time using GPUs. As no PCR amplification step is necessary and the detector can discriminate between methylated and unmethylated bases, HiFi sequencing can also be used to determine methylation status.

Pacific Biosciences provides PacBio Cloud and SMRT LINK software to analyze PacBio data and calculation of consensus sequences, and tools such as Racon and Pilon [19] can be used for further refinement. As with Nanopore sequencing, reads can be assembled using Canu [20] and Flye [21] or aligned to a reference genome using the minimap2-based pbhmm2. Long-read sequencing can also be used to improve transcriptome annotation by analyzing full-length isoforms and detecting rare transcripts using tools such as SQANTI3 [22], IsoSeq3/cDNA_Cupcake, and TAMA [23]. Long-read sequencing opens up new possibilities, such as matching the closest HLA allele using Hifihla, identifying methylated bases using mCaller [24], and detecting structural variants using pbsv or Sniffles [25].

##### Nanopore Sequencing

Nanopore sequencing from Oxford Nanopore Technologies solves many of the same problems as PacBio sequencing but works instead by measuring the changes in an electrical field as a DNA sequence passes through a microscopic pore. This method is less precise than short-read sequencing but produces much longer reads, between 10 kilobases and 1 megabase under certain conditions. Nanopore sequencing can also be performed using inexpensive flowcells on small portable devices such as MinION, which has contributed greatly to efforts to monitor SARS-CoV-2 [26,27]. Despite these advantages, long-read sequencing is unlikely to supplant short-read sequencing, and hybrid methods provide the best of both worlds by using ultra-long-read sequencing for scaffolding paired with short-read mapping to improve accuracy.

Long-read sequencing requires different approaches and software than short-read sequencing. Base-calling the raw output from Nanopore sequencers requires specialized tools, such as Guppy and Bonito. Quality control software such as NanoPack [28] and porechop [29] contain methods for plotting and filtering long-read data and removing chimeric reads. Canu [20] and Flye [21] perform de novo assembly, and Minimap2 [30] and NGMLR [25] map reads to a reference genome. The higher error rate is a known limitation of Nanopore sequencing, but error correction tools like Nanopolish [31] and Medaka make use of information contained in the raw signal data to recalibrate base calls and polish assemblies. Medaka can also be used for variant calling, along with tools such as Longshot [32], Pepper-Margin-DeepVariant [33], and Sniffles [25].

#### 2.1.5. Public Repositories of Genomic Data

The most consequential benefit of the Human Genome Project is that the findings were made public and can be freely accessed by anyone anywhere in the world. This is a remarkable achievement and sets an important precedent for democratizing access to biological data. The Genomic Standards Consortium is an open-membership organization tasked with overseeing and guiding the stewardship of genomic data along with the biological context [34].

##### DNA Sequence Databases

Most genomic data in the public domain are managed by the International Nucleotide Sequence Database Collaboration (INSDC) [35], which includes GenBank, the European Molecular Biology Laboratory (EMBL) Nucleotide Sequence Database, and the DNA Data Bank of Japan (DDBJ). Sequence data are carefully annotated and cross-referenced and can be queried and exported in multiple ways. The University of California, Santa Cruz (UCSC) Genome Browser also provides useful tools for genome visualization with fine-grained control over track inclusion and formatting.

##### DNA Variant Databases

The human reference genome is a haploid sequence that does not represent the genome of any single person but serves as a coordinate system and point of reference. A number of projects have attempted to assess the range of variation among individuals within and among populations, including the Human HapMap project [36], the 1000 Genomes project [37], the UK Biobank [38], and All of US [39]; the last of which aims to include a diverse group of more than a million people in the United States. Information gleaned from these databases is widely used in genome-wide association studies to identify germline variants associated with disease risk, response to treatment, personalized medicine, etc. [40]. In cancer genome profiling, tools such as Mutect2 [41] can be used to identify somatic variants that appear in paired tumor-normal sequencing and that are uncommon in the general population. Packages such as SnpEff [42] can be used to predict the potential effect of single nucleotide variants on coding sequences and compared to known variants by querying databases such as ClinVar [43].

### 2.2. Epigenomics

Many bioinformatics studies compare gene expression levels in samples under different conditions using RNA sequencing or transcriptomics, but fundamental insights may also be gained by examining the epigenetic modifications such as DNA or histone methylation or histone acetylation that influence the extent to which a gene is accessible to transcription machinery within the target sample. Epigenetic regulation is a crucial aspect of developmental biology, and epigenetic dysregulation is a common characteristic of many diseases such as cancer and is important in the development of novel treatments [44].

An advantage of Nanopore sequencing is that the passage of methylated DNA through the nanopore opening creates a signature that can be interpreted from raw signal data using tools such as Nanopolish [31] and Tombo [45]. However, a number of dedicated epigenomics methods have also been developed, including bisulfite sequencing and methylated DNA immunoprecipitation sequencing to characterize patterns of DNA methylation and ATAC-Seq, DNase-seq, and ChIP-seq to identify regions of chromatin accessible to transcription machinery. Tools for epigenomics analysis include Bismark [46] and MethylKit for bisulfite sequencing [47], MACS [48] for ChIP-seq data, and HMMRATAC [49] and Signac [50] for chromatin accessibility studies. The Encyclopedia of DNA Elements (ENCODE) project is an ambitious international collaboration that set out to catalog the functional elements within the human genome [51]. The UCSC Genome Browser includes a number of ENCODE tracks including transcription factor binding sites, DNase hypersensitivity, and histone marks that can aid interpretation of gene expression data.

### 2.3. Transcriptomics

Genomics provides invaluable insight into genetic variation among individuals as well as within heterogeneous tissue such as tumors, but the information only reveals an organism’s genetic potential. The transcriptome provides much more direct insight into which genes and transcripts are being actively expressed. Cells contain an enormous amount and variety of RNA, including messenger RNA, ribosomal RNA, transfer RNA, micro-RNA, small nuclear RNA, small nucleolar RNA, PIWI-interacting RNA, long-non-coding RNA, etc. As only mRNA and miRNA are of interest in most studies, this large excess of RNA poses a challenge and may be removed prior to sequencing. Mapping mRNA back to the genome for identifying and counting also poses a challenge due to splice junctions. Tools such as Salmon [52] skirt this problem by mapping reads to a transcriptome consisting of known transcripts, but this method is only suitable for known splice junctions. Popular splice-aware tools such as STAR [53], Tophat2 [54], and HISAT2 [55] map reads directly to the reference sequence based on genome annotations and can be used to detect novel transcripts.

A major goal of transcriptomics is to compare gene expression under different conditions. As a sequencing run acts as a batch effect, it is advantageous to run as many samples as possible within the same sequencing run. Barcoding makes this possible by including a short lane-specific barcode sequence within the adaptor. During the quality control stage, sequence reads are first demultiplexed and labeled. Popular differential gene expression tools such as Limma-voom [56], DEseq2 [57], and edgeR [58] compare expression levels by sampling each transcript and calculating a *p*-value and fold-change. The tools vary in detail, but each tool takes into account factors such as library size, RNA length, and multiple testing to minimize confounding factors. However, RNA-Seq is a relative measure, and it is difficult to compare expression levels across experiments. RNA-Seq also provides insight into alternative splicing using tools such as rMATS [59] and DEXSeq [60].

Aside from reporting up- and down-regulated genes, many studies perform some form of pathway or functional analysis using tools such as GSEA [61], DAVID [62], or Panther [63] to identify overrepresented pathways or gene ontology terms. Pathway data can be visualized using KEGG Mapper [64] and Cytoscape [65]. Unsupervised methods can also provide powerful insight into RNA-Seq data. Dimension reduction techniques such as PCA, t-SNE, and UMAP help to identify structure within the data and reveal outliers. Weighted gene correlation network analysis using WGCNA [66] uses hierarchical clustering to classify genes into clusters and identify eigengenes or hub genes associated with experimental conditions, which can serve as targets for further experimentation.

Several public repositories have been established to store RNA-Seq data, and tools such as Gene Expression Omnibus [67] and ArrayExpress [68] accept experimental data to facilitate differential gene expression analysis.

### 2.4. Proteomics

While transcriptomics is essential for elucidating regulatory pathways, it is misleading to assume that gene expression levels accurately reflect the actual protein levels. In mammals, only about 30–40% of the variation in protein levels is associated with corresponding mRNA levels, highlighting the importance of post-transcriptional and post-translational regulation [69]. The goal of proteomics is to identify and quantify the proteins present in a sample as well as to determine protein structure, function, and interactions [70]. Cells are lysed, and proteins are extracted and digested into smaller peptides. Proteins can be separated based on properties such as charge and mass using methods such as gel electrophoresis and liquid chromatography. Peptides are ionized using electrospray ionization or matrix-assisted desorption/ionization and then separated based on the mass-to-charge ratio in a mass analyzer. Proteins are identified and quantified by comparing spectrum peaks against a database of theoretical spectra using software such as MaxQuant [71], MSstats [72], and PRIDE (Proteomics Identification Database) [73]. As with gene expression data, differential protein abundance data can be analyzed using pathway and functional analysis tools such as KEGG [64], DAVID [62], and Reactome [74]. Protein–protein interaction networks can be analyzed using STRING [75].

#### 2.4.1. Public Repositories for Protein Data

Analogous to the Genomics Standards Consortium for genomics data, the Proteomics Standards Initiative [76] establishes community standards for the management of proteomics data, including the exchange of mass spectrometry and protein–protein interaction data. UniProt is the primary public database for protein sequence data and contains both automatically annotated sequences in UniProtKB/TrEMBL as well as expertly curated and experimentally verified protein data in UniProtKB/Swiss-Prot. The Human Protein Atlas is another important database dedicated to mapping all human proteins by organ, tissue, and cell type [77].

#### 2.4.2. Protein Structure Prediction

The three-dimensional structure of a protein is also critically important and is often only solved after laborious crystallization efforts spanning months or years. Solved protein structures are stored in the RCSB Protein Data Bank [78]. Although the structures of novel proteins can be predicted using homology modeling using tools such as SWISS-MODEL [79], accuracy is often low. Protein structure prediction has long remained an open challenge even with state-of-the-art tools, but this changed dramatically following the introduction of the advanced machine learning tool AlphaFold [80]. Predicted structures for over 214 million proteins have been deposited in the AlphaFold Protein Structure Database [81].

#### 2.4.3. Post-Translational Modifications

Analysis of protein data includes an additional layer of complexity due to the more than 650 known forms of post-translation modification, including phosphorylation, glycosylation, ubiquitination, methylation, acetylation, and SUMOylation [82]. Up to two-thirds of all proteins are thought to undergo phosphorylation, in which a phosphate group is reversibly added to a serine, threonine, or tyrosine residue [83]. Phosphoproteomics is an important branch of proteomics that can provide additional insight into protein function, such as identification of drug targets within signal transduction pathways. Phosphoproteins in the sample are enriched using affinity purification followed by mass spectrometry [84].

### 2.5. Glycoinformatics

More than half of all proteins are also thought to be glycosylated, in which a sugar moiety called a glycan is attached to an asparagine (N-linked glycoproteins) residue or to a serine or threonine residue (O-linked glycoproteins) [85]. Glycosylation plays underappreciated roles in protein folding, cell–cell interactions, and immune regulation, and aberrant glycosylation has been implicated in autoimmune diseases and cancer [86].

Glycomics is the study of the diversity of glycan structures, while glycoinformatics is a branch of bioinformatics tasked with the storage, visualization, and analysis of glycan data. Unlike genes and proteins, glycans cannot be encoded directly as a linear sequence but rather are formed by the coordinated activity of numerous glycosyltransferases in different compartments within the cell.

Glycan structures are determined by releasing, labeling, and separating glycan chains followed by mass spectroscopy or nuclear magnetic resonance spectroscopy and comparing spectra with known glycan databases such as GlyTouCan [87] and UniCarbKB [88]. Lectin arrays are another approach to glycan identification, which takes advantage of the high binding affinity and specificity of lectins for specific glycan structures [89]. A number of databases and tools attempt to integrate glycan data, including GlycoPOST [90], the GlyCosmos Portal [91], GlyGen [92], and Glycomics@Expasy [93].

### 2.6. Metabolomics

If a cell’s DNA represents the backdrop and the RNA and proteins the actors, then the cell’s metabolites are the dialogue of a given scene. Metabolomics is the comprehensive analysis of metabolites in a specimen, yielding unprecedented insight into internal biochemical activity and “chemical fingerprints” of cellular processes [94]. Metabolites are small molecules that often serve as intermediates or end products in metabolic pathways, including simple sugars, fatty acids, amino acids, nucleotides, vitamins, and organic acids. Of particular concern in capturing metabolomics data is the rapid collection and storage of samples to preserve the state of metabolic reactions. A combination of polar and non-polar solvents, filtration, centrifugation, separation by chromatography or electrophoresis, and chemical modification to improve volatility or stability may be needed to extract biochemically diverse metabolites. Identification involves mass spectrometry or nuclear magnetic resonance and comparison of peaks against known spectra and annotation using databases such as the Human Metabolome Database [95] and the Small Molecule Pathway Database [96]. Experimental data are also contained within the MetabolomeXchange [97] and MetaboLights [98] databases. MetaboAnalyst [99] and MetaboDiff [100] are popular tools for differential metabolomics analysis. As with similar initiatives for genomics and proteomics, the Metabolomics Standards Initiative was established to propose reporting standards and guide the development of repositories; however, the field of metabolomics is less mature, and reporting guidelines have not been followed closely [101,102].

#### Lipidomics

Lipidomics is a branch of metabolomics that focuses on lipids, such as fatty acyls, glycerolipids, glycerophosphospholipids, sphingolipids, saccharolipids, polyketides, sterols, and prenols [103,104]. Lipids are assembled from ketoacyl and isoprene groups and tend to form from molecules with either hydrophobic or amphiphilic properties. Due to their unique properties, lipids perform a number of important functions within cells. Aside from the essential structural role of phospholipids in forming cellular membranes, lipids serve as a stable high-yield energy store in the form of triglycerides. Lipids also act as signaling molecules, such as prostaglandins, leukotrienes, steroid hormones, phosphatidylinositol, diacylglycerol, and ceramide. As lipid dysregulation can affect metabolic regulation, cell signaling, survival, proliferation, inflammation, electrolyte balance, reproduction, and immune response, analysis of the lipid content of cells can provide important insight.

However, the unique and diverse properties of lipids also make them difficult to analyze. As one of the least mature omics fields, lipidomics best practices have yet to be agreed upon, although the Lipids Standards Initiative aims to establish minimum standards for reporting [105]. This effort is complicated by the diverse nature of lipids, as analysis of non-polar and polar lipids requires different approaches. For example, polar lipids such as phospholipids can be separated from non-polar lipids such as triglycerides using the modified Bligh and Dyer method followed by two-dimensional silica gel thin-layer chromatography or hydrophobic interaction liquid chromatography (HILIC) [106,107,108]. Lipids are identified via mass spectrometry using electrospray ionization or tandem mass spectrometry. Mass spectrometry findings are reported as spectra that must be matched to those of known lipids for identification using tools such as Lipostar [109], Mzmine [110], LipidBlast [111], MS-DIAL [112,113], LipidSearch [114], and LipidMaps [115]. Metabolites may be labeled with identifiers from HMDB, KEGG, and ChEBI, although lipids such as triacylglycerols, diacylglycerol, phosphatidylcholine, phosphatidylethanolamine may be sub-classified further based on the number of carbons and double bonds, e.g., PC(40:5).

Despite its importance, the field of lipidomics faces several unique challenges. Given the number of tools and the lack of established best practices, reproducibility and agreement among methods are of paramount importance, but von Gerichten et al. showed only 14% agreement between MS DIAL and Lipostar given the same LC-MS spectra [116]. They encourage the use of validation across positive and negative LC-MS modes and manual curation of the output to minimize false-positive identification.

### 2.7. Single-Cell RNA Sequencing

Bulk RNA sequencing is efficient in terms of the scale of sequencing but discards all information about spatial context and variation among cells of the same or different type [117]. This is particularly limiting in the analysis of complex samples such as blood and tumor samples.

Even with bulk sequencing, it has long been possible to use barcodes to separate reads based on lane, and single-cell sequencing extends this approach to label individual cells prior to sequencing [118]. First, samples are digested and filtered to isolate individual cells. Early methods involved distributing cells within wells on a plate, but newer methods capture cells using microfluidic droplets along with beads containing barcodes to identify the cell of origin. Amplified cDNA is sequenced together and then demultiplexed, providing simultaneous sequencing of tens of thousands of cells. As cells are sequenced individually using arbitrary identifiers, it is necessary to use dimension reduction techniques such as T-SNE or UMAP to identify cell clusters at a selected resolution for further analysis. Although scRNA-Seq is a powerful and increasingly widely used technique, isolating and labeling individual cells poses practical quality control challenges. Both the sample volume and the number of beads must be maintained at relatively low values to reduce the frequency of doublets (wells containing more than one cell or bead).

Despite efforts to reduce amplification bias, PCR amplification from a small initial sample can introduce bias related to GC content, fragment length, and stochastic effects, which can reduce confidence in quantitative analysis and detection of rare variants. Removal of PCR duplicates based on alignment coordinates does not prevent the removal of valid biological duplicates. One solution used in some single-cell sequencing methods involves the addition of a random 6–12 base pair oligonucleotide called a unique molecular identifier (UMI) prior to amplification [119]. This makes it possible to differentiate between PCR and biological duplicates, as all reads sharing the same UMI are considered to correspond to the same read. However, the use of UMIs introduces additional complexity and is not used in methods such as SMART-seq2 [120].

Although similar in principle to the tools for bulk RNA-Seq, scRNA-Seq has resulted in the development of numerous toolkits for analyzing single-cell data. scDblFinder can be used to remove doublets [121], and SingleCellExperiment [122] is a Bioconductor package that can be used to organize experimental data. UMI-tools [123] and zUMIs [124] provide tools for working with UMIs. Seurat [125] is a popular R library for single-cell, spatially resolved, and integrative multimodal analysis. In a typical Seurat workflow, scRNA-Seq data may be downloaded from a public repository, filtered interactively based on sequencing depth, level of mitochondrial DNA, and cell-to-cell variability, normalized and scaled, assigned to clusters, and prepared for differential gene expression analysis. Similarly, SCANPY is a Python toolkit for analyzing scRNA-Seq data and includes methods for preprocessing, visualization, clustering, and differential expression analysis [126]. Although single-cell methods are not able to incorporate spatial data, tools such as Monocle3 [127] can analyze trends over “pseudo-time” using trajectory analysis, useful in studies involving stem cells, for example, in which changes in gene expression profiles among cells reflect underlying transitions in state.

Chromatin accessibility can also be analyzed at single-cell resolution using single-cell assay for transposase-accessible chromatin sequencing (scATAC-seq) with support from tools such as Signac [50], SnapATAC [128], Scregseg [129], and ArchR [130].

### 2.8. Spatially Resolved Transcriptomics

Even though single-cell RNA sequencing provides high-resolution data at the level of individual cells, it shares one of the same limitations of bulk RNA sequencing: it discards information about the spatial orientation of cells. In some cases, this can affect interpretation, such as when examining the tumor microenvironment [131], and in tissues such as liver sinusoids, in which hepatocyte activity varies across a gradient from the portal vein to the central vein [132]. Spatially resolved transcriptomics is a flexible technique that can be used to compare gene expression at the level of small clusters of cells, at single-cell resolution, and even subcellular resolution to reveal variation in transcript abundance within different regions of a single cell. Several methods have been developed to incorporate spatial data into the gene expression analysis. For example, RNA molecules can be directly labeled within tissue sections using multiplexed error-robust fluorescence in situ hybridization [133]. Alternatively, tissue samples can be mounted to a glass slide with the spatial coordinates of each region encoded within barcode adapters using the 10x Genomics Visium platform. Hybrid techniques such as Nanostring GeoMX can simultaneously profile RNA and proteins using oligonucleotide tags. Seurat and Scanpy can also be used to analyzed spatially resolved transcriptomics data, and a number of other R and Python tools and frameworks have been developed, including Squidpy [134], Giotto [135], SPATA2 [136], SpatialDE [137], and BayesSpace [138]. Single-cell and spatial bioinformatics still face a number of hurdles in terms of cost and complexity and the need for specialized equipment and workflows. Bulk sequencing may yield better results for homogeneous or delicate samples as well as for examining novel transcripts and genes with low levels of expression, but adoption of single-cell and spatial methods will continue to grow.

### 2.9. Radiomics

One of the most exciting areas of AI adoption in bioinformatics is radiomics, in which deep learning features extracted from medical imaging data such as MRI, CT, and PET are combined with clinical data and other omics data to yield unprecedented insight into disease and clinical outcomes [139]. The goal of radiomics is to identify quantifiable and reproducible features from images or series of images, such as changes in the shape and heterogeneity of a lesion over time [140]. Features can correspond to either two- or three-dimensional regions of interest and can be based on first-order single-pixel or single-voxel gray levels or can take into account texture gradients within the image, such as the gray-level cooccurrence matrix, the neighborhood gray-level, or more complex shapes. Methods such as Fourier transformation and wavelet transformation can also be used to facilitate segmentation and edge detection prior to analysis. Development in this area is rapid, and tools such as CelloType provide end-to-end models that perform both segmentation and classification [141]. Radiomics faces pitfalls due to the need for a large number of high-quality images obtained under homogeneous conditions without artifacts. Class imbalance and over-fitting are also perennial challenges in the training of supervised deep-learning models. The Image Biomarker Standardization Initiative screened a set of 174 radiomic features from CT, PET, and MR imaging and standardized on a set of 169 features with high reproducibility [142].

## 3. Methods and Tools for Integration and Joint Analysis of Multi-Omics Data

**Figure 2:**
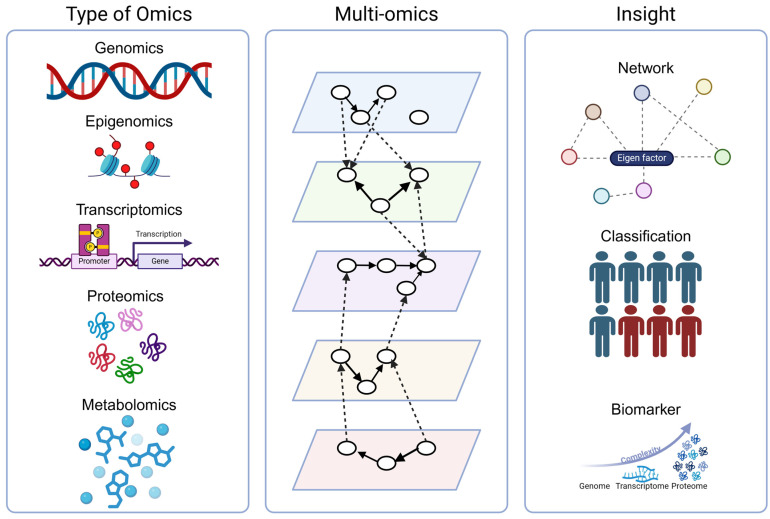
Multi-omics concepts. Each distinct omics method provides information about a different but incomplete aspect of the internal state of the cell. Joint analysis of two or more omics methods provides more comprehensive insight into key factors that can be used for classification or prediction and or serve as potential biomarkers or drug targets. Created in part using BioRender.com (accessed on 25 November 2024).

In the transition from microarrays to RNA-Seq, differential gene expression data are often reported alone as a single-omics study, whereas studies incorporating alternative omics methods such as proteomics and metabolomics often anticipate the logical follow-up question of how observed changes relate to underlying patterns of gene expression. However, researchers may be uncertain how to combine multi-modal data and effectively end up reporting them as standalone results. The introduction of single-cell and spatially resolved methods has further exacerbated this difficulty by including additional levels of information that should be taken into account. The challenge of multi-omics is that adding more data is just as likely to obfuscate the underlying signal as it is to clarify it. In the ideal case of vertical integration, multiple forms of omics data are collected from the same samples, but it should also be possible to analyze data collected using different samples (horizontal integration) or even different types of omics methods on different samples (diagonal integration). Regardless of which omics methods are employed or how they are integrated, multi-omics analysis provides a sophisticated multi-layered framework that can be used to identify key drivers and pathways as well as to develop classification and prediction models (Figure 2). An overview of the major types of omics data and associated tools is shown in Table 2.

### 3.1. Statistical Approaches to Multi-Omics Analysis

There are many approaches to the integration of multi-omics data, but common themes include feature selection using methods such as LASSO and elastic net, as reviewed in Wu et al. [143], and extraction of higher-level representations using dimension reduction techniques. Methods such as hierarchical clustering, K-nearest neighbors, t-SNE, and UMAP help to simplify the analysis and visualization of high-dimensional data and reveal clusters and outliers. The naïve approach to integrating different forms of omics would be to treat the combined data as additional columns in the table in the same way as increasing the number of probes on a microarray, but this approach has a number of statistical pitfalls due to the different types of data collected, which will not initially be easily integrated. However, latent factor analysis using methods such as canonical covariance analysis (CCA) and non-negative matrix factorization (NMF) provides a statistically sound way to integrate different forms of data by reducing the dimensionality of each data set into representative factors based on joint and omics-specific sources of variation. For example, CCA maximizes the correlation among linear combinations of variables between two data sets, while the canonical correlations indicate how much of the variation in one form of omics data is explained by the other. Regardless of the method, preprocessing is an essential step and may involve normalization, scaling, dimension reduction, imputation, and filtering. Preprocessing tools are often included with the analysis frameworks but are also available as standalone tools such as MultiAssayExperiment and OmicsData. While an examination of differentially expressed genes or proteins/metabolites can be used to identify biomarkers in single-omics analysis, an advantage of CCA is that canonical loadings can be used to identify biomarkers that are informative across molecular levels.

### 3.2. Partial Least Squares Integration Using MixOmics

MixOmics implements a number of vertical and horizontal multi-omics integration methods as well as offers guidance on how to select the most appropriate approach depending on the goals and limitations of the study [144]. For example, the multivariate integration (MINT) framework can be used to combine multiple data sets that involve the same omics method. Multigroup sparse partial least squares (sPLS) is an unsupervised method, whereas multigroup sparse partial least squares discriminant analysis (sPLS-DA) is a supervised method used for classification. For vertical integration with multi-omics data collected from the same samples, multiblock sPLS can be used for unsupervised analysis, while multiblock sPLS-DA (nicknamed DIABLO) is suitable for supervised analysis. The mixOmics website provides example data sets, including a case study on breast cancer using mRNA, miRNA, and proteomics data, as well as case studies involving ABC transporters, microbial communities, and acetaminophen toxicity in rat livers. MixOmics has also been used as the primary analysis tool in a number of studies. For example, Jiang et al. used mixOmics to identify a long non-coding RNA implicated in breast cancer proliferation [145], while Hu et al. used mixOmics with transcriptomics and metabolomics data for ^13^C metabolic flux analysis in *Bacillis subtilis* [146]. We also used mixOmics in a study of fatty liver in a human hepatocyte chimeric mouse model [147].

### 3.3. Multi-Modal Weighted Correlation Network Analysis with MiBiOmics

However, this table-based method is by no means the only approach to integrating multi-omics data. MiBiOmics [148] extends the weighted gene correlation network analysis concept in the WGCNA package to support both proteomics and metabolomics data sets. Eigen-genes, eigen-proteins, and eigen-metabolites constitute a form of dimension reduction within each omics type by highlighting genes, proteins, and metabolites that best represent a cluster of highly correlated markers within each data set. MiBiOmics then takes this a step further by examining the correlations among each set of eigen-markers, revealing cross-omics patterns. The method is unsupervised but modules can be plotted with user-provided sample data to identify potentially interesting biomarkers. The method also helps to visualize the relationship between omics source and class data and uses multiple co-inertia analysis to identify highly influential biomarkers [149].

### 3.4. Pathway-Based Multi-Omics Analysis

Nonetheless, a key limitation of these methods is that they ignore the biological context. There are benefits to detecting correlations among cross-omics biomarkers based only on count data, but other approaches seek to take into account known functional associations between biomarkers. Tools such as Pathway Commons, PaintOmics [150], and Cytoscape plugins such as cytoscapeOmics and OmicsNet support multi-omics analysis based on biological pathways [151].

### 3.5. Cloud-Based Tools

Of the multi-omics tools discussed so far, MiBiOmics has an advantage in ease of use as a web-based tool. Laboratories often outsource omics data collection and receive a detailed, high-quality analysis of the single-omics results. However, the researcher may then struggle to combine the findings into a unified multi-omics analysis and may look to web-based tools as a first step before committing to local tools with a steeper learning curve. However, MiBiOmics is not particularly intuitive and requires data to be uploaded in a specific format. It also lacks persistent sessions, a distinct disadvantage in the case of long-running analyses. Galaxy has long been used for online analysis of bioinformatics data and includes workflows for quality control, integration, and analysis of multi-omics data (Galaxy 2024). Mergeomics is another web-based tool but is oriented more toward disease-association studies such as genome-wide association studies, transcriptome-wide association studies, etc., to identify key drivers and predict drug targets [152]. OmicsNet [153] is a network-based tool that provides 2D and 3D graph layouts and provides support for SNP data, microbiome profiling taxon data, and metabolomics LC-MS peaks data. PhenoMeNal [154] is a cloud-based multi-omics platform with support for metabolomics data. The Analyst Suite is a recent multi-omics extension of the popular MetaboAnalyst metabolomics tool [155]. Commercial tools include the Illumina BaseSpace Sequence Hub and Correlation Engine, the Seven Bridges Genomics platform, Google Cloud Life Sciences, AWS Genomics, Microsoft Azure for Genomes, and DNAnexus Apollo.

### 3.6. Locally Installed and Desktop Multi-Omics Tools

Moving away from the cloud, the installation and use of multi-omics software on the desktop becomes more challenging and/or expensive, although several commercial desktop suites are available, including CLC Genomics Workbench, Partek Genomics Suite, DNASTAR Lasergene, Agilent GeneSpring, OriginPro, and SIMCA. However, aside from the licensing costs, the use of commercial desktop tools often requires a powerful desktop computer or GPU-equipped workstation and limits portability and collaboration. Commercial tools provided by the omics vendor likely provide access to features not available in open-source tools, but such tools are also likely to lag behind the rapid pace and innovation of open-source software. OmicsSuite aims for this middle ground with an open-source desktop application that provides an interface for 175 analysis tasks and integrates more than 300 R and BioJava packages [156].

### 3.7. Libraries and Command Line Tools

Over time, bioinformaticians are likely to assemble ad hoc pipelines ranging from RMarkdown or Jupyter notebooks to Docker containers, Bash scripts, and Snakemake or Nextflow workflows running on AWS or a cluster or supercomputer orchestrated using a grid engine like SLURM. In short, bioinformaticians often prefer using open-source Linux command line tools and libraries written in C/C++, R, Python, Matlab, Perl, or Java that can be chained together and automated in a flexible and reproducible way.

In this context, mixOmics, MiBiOmics, Mergeomics, and OmicsNet all provide R packages or source code, allowing them to be run as part of a workflow, and a vast number of other libraries and packages have also been published. Among the most popular are MOFA2 [157] for multi-omics analysis and MOFA+ [158], which supports single-cell analysis. MOFA2 and mixOmics are well suited for large data sets, whereas tools such as tidyomics integrate well with an R tidyverse workflow [159], and Anvi’o provides an integrated Python-based ecosystem for microbiome analysis [160]. As there is a vast range of tools at various stages of maturity, development activity, and community support, it is worth evaluating several different tools, especially those based on different analytical methods. For example, OmicsIntegrator [161] is network-based and useful for identifying pathways and key drivers, whereas MOGSA [162] uses generalized singular value decomposition to identify correlated low-dimensional features across omics types. IMPALA [163] and SMITE [164] are well suited for investigating biological mechanisms, while Omics Notebook [165] and Omics Playground [166] are low-code interactive platforms for exploratory analysis and visualization. NetZoo [167] is geared more toward the elucidation of gene regulatory networks and systems biology. MUON [168] is intended for single-cell data, while MEFISTO [169] and JIVE [170] support spatial and time-series data. A number of tools employ machine learning for prediction, including MOMA [171], Miodin [172], DeepMO [173], and MOGONET [174]. MultiDGD takes an innovative approach by incorporating a deep generative model to identify statistical associations between genes and regulatory regions using transcriptome and chromatin accessibility data [175]. Tools such as IMPALA have more complex installation requirements, and MOMA, DeepMO, and JIVE have greater computational demands and may require a GPU.

### 3.8. Public Repositories Containing Multi-Omics Data

It is safe to assume that data submitted to public repositories contain many interesting findings yet to be uncovered. A number of databases now provide well-annotated data for two or more types of omics methods that can be used for multi-omics analysis, including the ENCODE database, the Cancer Genome Atlas (TCGA) [176], the Human Cell Atlas, the Human Protein Atlas, Gene Expression Omnibus, the Proteomics Identifications Database, ArrayExpress, LinkedOmics, the 1000 Genomes Project, the Clinical Proteomic Tumor Analysis Consortium (CPTAC), the Molecular Taxonomy of Breast Cancer International Consortium (METABRIC), and the CellMiner NCI-60 cell line database [177].

### 3.9. Statistical Issues in Multi-Omics

Omics and multi-omics analysis often involves performing many thousands of statistical tests on high-throughput data and, by nature, is usually not designed with testing of specific a priori hypotheses in mind. Therefore, there is a severe risk of detecting false positives due to noise or random chance. New statistics students tend to assign undue importance to α = 0.05, where α is the Type I error, i.e., the probability of rejecting a null hypothesis when it is true. While this may or may not be reasonable for a single statistical test, it nearly ensures at least one false positive as the number of tests increases. There are several ways to mitigate this risk. Ideally, the number of replicates should be selected to provide sufficient power. Utilities such as the MultiPower tool [178] and careful reading of the documentation can help to reduce within-group variance and provide insurance against outliers, but practical limitations tend to dictate the number of replicates that can be included. Cost and complexity increase quickly with each additional omics method performed on a given set of samples.

The simplest approach to reduce false positives is to control the family-wise error rate by dividing α by the total number of tests being performed (Bonferroni correction). This conservative approach is often used when the risk due to false positives is high, such as in clinical trials and in genome-wide association studies. A related approach is the alpha-spending function, which can be used to help control the overall Type I error rate during interim analysis of a clinical trial [179]. However, there is a tradeoff between Type I and Type II errors, and Bonferroni correction reduces statistical power and increases the risk of false negatives [180]. As omics studies often represent the first stage in screening for potential drug targets or biomarkers, less stringent methods to control the false discovery rate (FDR) may be considered. Instead of attempting to prevent any false positives, FDR sets a limit on the tolerated proportion of expected false positives. FDR is often reported as a q-value, which is essentially a *p*-value adjusted for multiple testing. The two major methods to control FDR are Benjamini–Hochberg (BH) and Benjamini–Yekutiele (BY), which differ mainly with respect to assumptions of independence among tests [180].

However, assumptions of independence as well as assumptions concerning the underlying distribution, homoscedasticity, etc., are another area of concern in the analysis of omics data. Some methods are relatively robust to minor violations of the assumptions, but these problems are exacerbated in high-dimensional studies over a typically small number of samples. Exploratory analysis should be conducted prior to analysis to ensure that data are normalized and standardized appropriately and that missing values are imputed or handled as necessary. Examination of control data should help to highlight batch effects and discriminate between technical and biological sources of variation. Outliers and influential points should be investigated, and multicollinearity among features should be addressed. The intersection of different types of omics data is particularly complex in this regard, and important relationships should be plotted to determine whether the pattern is biologically meaningful or a statistical artefact. There is little benefit in attempting to include all available single-omics features in a multi-omics analysis, and many tools recommend removing sparse or low-variance features prior to analysis.

However, despite these technical concerns, the most important consideration is understanding the limits on what can be asked of a given data set, and this is largely a matter of discipline and preparation. For example, genome-wide association studies provide an excellent starting point for future studies, but little more can be asked of the findings than that, as is often borne out by failure to replicate initial findings in follow-up studies in a different population. Therefore, omics data should be used mainly to generate hypotheses that are then tested using independent data. A posteriori testing and post hoc theorizing are similar to the concept of overfitting in machine learning and increase the Type I error rate. Hypothesizing After the Results are Known [181,182], or HARKing, leads to poor reproducibility and is simply bad science. With great power comes great responsibility, but considering the small sample sizes used in most multi-omics studies, perhaps this should be amended: With little power comes even greater responsibility.

## 4. Discussion

There is no guarantee that gathering more and different types of data will make a problem easier to solve, and rapid expansion of the omics portfolio brings with it a new set of challenges [183]. A selection of well-executed multi-omics studies is shown in Table 3, but many other studies are likely to either become bogged down in analysis paralysis or move to publish too quickly after a superficial analysis. Both cases represent missed opportunities that tool developers should strive to help avoid by focusing on streamlining the most common use cases and identifying unmet needs among end users. However, while making tools easier and more robust to use will benefit users, ease-of-use is less important than giving users confidence in being able to use the tools and interpret the results, especially when challenged during peer review or a doctoral examination. Open-source tools are much less likely to provide up-to-date tutorials, realistic sample data, walkthroughs, cookbooks, videos, frequently asked questions, etc., in the way that commercial tools do, nor are they as likely to include rigorous testing and user input validation to ensure that user data satisfy assumptions made by the software. This is unfortunate as these tools often represent the cutting edge and are sometimes provided as reference implementations of novel algorithms that are eventually incorporated into commercial tools.

Tool developers could also help to improve the design of experiments by helping to guide the user regarding sample size and the optimal number and variance of features. Multi-omics analyses can also be waylaid by misunderstandings regarding nomenclature and ID mapping among databases. Tools can help reduce this risk by providing flexible data import options and sanity checks to help users assess whether the mapping is appropriate. Despite being the most mature omics area, or perhaps because of it, this problem affects genomics and therefore transcriptomics most acutely. Common names should be discouraged in favor of HUGO Gene Nomenclature Committee (HGNC) names, Entrez Gene IDs, Ensembl IDs, or other well-maintained identifiers. Bioconductor and Ensembl BioMart provides excellent support for converting between identifiers, and providing this function within the tool can help reduce user error. However, arbitrary identifiers make interpretation more difficult, and many genes are more commonly known under a common alias than the official HGNC name, so some flexibility should be considered in displaying genes and other features for exploratory analysis and publication.

Multi-omics data are expensive and difficult to generate and analyze, and the development of new tools will likely be able to tease out patterns that are missed by current tools. Therefore, journals should strongly encourage the submission of multi-omics data to public repositories whenever possible. Developing integrated tool suites and well-supported best practices similar to the GATK should help researchers publish meaningful and reproducible studies. In addition to enforcing minimal reporting guidelines, repositories should strive to follow FAIR Guiding Principles (Findability, Accessibility, Interoperability, and Reusability), which helps to automate machine-finding and reuse of data [200].

## 5. Conclusions

The future of bioinformatics appears encouraging, with rapid innovation in the diversity and resolution of omics data and the development of new analysis tools. The next stage will likely see rapid growth of multi-omics databases and greater flexibility for end users through cloud-based tools based on open-source R and Python packages and improved reproducibility and scalability using Docker containers, Jupyter notebooks, and workflow scripts. Considering the enormous impact that artificial intelligence and deep learning methods have had in nearly every field, the use of AI in bioinformatics is also increasingly widespread, marking important advances in gene prediction, variant calling, literature mining, and drug discovery [201]. The adoption of deep learning approaches may be difficult to distinguish from more traditional multivariate analysis methods already in use, but the breakthroughs achieved by AlphaFold2 will likely inspire a rapid wave of innovation in this area. Ultimately, the goal of multi-omics is to provide a more nuanced insight into the incredible complexity of the world within the cell. At first glance, the image appears obscure and incomprehensible, but like shadows on a screen, it is often a matter of perspective.

## Figures and Tables

**Table 1:** Comparison of first-, second-, and third-generation sequencing technologies.

Generation	First-Generation	Second-Generation	Third-Generation	
Platform	Sanger	Illumina	PacBio	Oxford Nanopore
Year	1987	2006	2009	2015
Sequencing technology	Chain termination method	Sequencing by synthesis	Circular consensus sequencing	Electrical detection
Current read length	800–1000 bp	100–300 bp	10,000–25,000 bp	10,000–30,000 bp
Current throughput	low	high	high	moderate
Current cost	low	moderate	high	high
Current read accuracy	high	high	moderate	low
Difficulty of analysis	low	moderate	high	high
Computing requirements for analysis	low	high	high	high

**Table 2:** Major omics types and analysis tools.

Omics	Description	Tools
Genomics for short reads	Analysis of the genome or exome using short paired or unpaired reads.	FastQC, Trimmomatic, AfterQC, fastp, BWA, GATK, SnpEff, SnpSift, ANNOVAR, Ensembl VEP, MultiQC, CNVkit, DNAcopy, manta, DELLY2
Genomics for long reads	Analysis of the genome using much longer read lengths, supports haplotype analysis and sequencing of highly redundant regions	NanoPack, porechop, Minimap2, NGMLR, Canu, Flye, Nanopolish, Medaka, Longshot, Pepper-Margin-DeepVariant, Sniffles
Epigenomics	Analysis of DNA methylation and other epigenetic modifications	Bismark, MethylKit, MACS, HMMRATAC, Signac, DESeq2, edgeR
Transcriptomics	Analysis of gene expression levels based on transcribed RNA levels	FastQC, Trimmomatic, AfterQC, fastp, STAR, HISAT, HTSeq-count, RSEM, kallisto, Salmon, featurecounts, StringTie, Ballgown, DESeq2, edgeR, rMATS, DEXSeq
Proteomics	Analysis of the proteins present within cells, possibly including analysis of post-translational modifications	ProteoWizard, MaxQuant, Msstats, OpenMS, DESeq2, edgeR
Glycomics/glycoinformatics	Analysis of the diversity of glycan modifications within cells	Glycoworkbench, MALDIquant, nQuant
Metabolomics and lipidomics	Analysis of small molecules and metabolites within cells	xcms, MetaboAnalyst, MetaboDiff, metabolomicsR, MS-DIAL, LIPID MAPS
Single-cell RNA sequencing	Transcriptomics resolved at the level of individual cells but without regard for spatial orientation	FastQC, Trimmomatic, AfterQC, fastp, STAR, HISAT, Cell Ranger, Seurat, Scanpy, Harmony, SingleR, Garnett, edgeR, DESeq2
Spatially resolved transcriptomics	Analysis of cells with respect to their spatial orientation	FastQC, Space Ranger, Squidpy, Giotto, Seurat, BayesSpace
Radiomics	Analysis of medical imaging data	pyradiomics, 3D Slicer, ImageJ/Fiji
Multi-omics	Joint analysis and integration of two or more types of omics data	mixOmics, MiBiOmics, MOFA2, MOFA+, IMPALA, NetZoo, MEFISTO, FactoMineR, MOVICS, PaintOmics, OmicsNet, OmicsSuite, Mergeomics, PhenoMeNal, Analyst Suite, Illumina BaseSpace Sequence Hub, Seven Bridges Genomics platform, Google Cloud Life Sciences, AWS Genomics, Microsoft Azure for Genomes, and DNAnexus Apollo, CLC Genomics Workbench, Partek Genomics Suite, DNASTAR Lasergene, Agilent GeneSpring, OriginPro, SIMCA

**Table 3:** Selected multi-omics studies and the omics methods included (indicated using a checkmark).

Study	RNA-Seq	scRNA-Sq	WGS/WES	Proteome	Metabolome	Lipidome	miRNA	Spatial Transcriptome
Chen et al. [184]	✓	✓		✓				
Lu et al. [185]		✓						✓
Ganguly et al. [186]	✓			✓				
Maan et al. [187]	✓				✓	✓		
Zheng et al. [188]	✓		✓	✓	✓		✓	
Ye et al. [189]	✓							✓
Xu et al. [190]	✓		✓					
Overmyer et al. [191]	✓			✓	✓	✓		
Braytee et al. [192]	✓		✓				✓	
Holý et al. [193]	✓		✓				✓	
Lee et al. [194]				✓	✓	✓		
Lv et al. [195]		✓						✓
Jia et al. [196]	✓						✓	
Kim et al. [197]		✓						✓
Ruan et al. [198]	✓	✓						
Ding et al. [199]			✓	✓	✓	✓		

## Data Availability

No new data were created or analyzed in this study. Data sharing is not applicable to this article.
